# Contribution of Obesity to Polypharmacy in U.S. Older Adults

**DOI:** 10.1007/s11606-026-10458-8

**Published:** 2026-06-04

**Authors:** Alissa S. Chen, Ashwin K. Chetty, John A. Batsis, Kasia J. Lipska, Alexandra M. Hajduk

**Affiliations:** 1 Section of General Internal Medicine, Department of Internal Medicine, Yale School of Medicine, New Haven, CT USA; 2 Yale School of Medicine, New Haven, CT USA; 3 Division of Geriatric Medicine and Center for Aging and Health, University of North Carolina at Chapel Hill, Chapel Hill, NC USA; 4 Department of Nutrition, Gillings School of Global Public Health, University of North Carolina at Chapel Hill, Chapel Hill, NC USA; 5 Center for Outcomes Research and Evaluation, Yale-New Haven Health System, New Haven, CT USA; 6 Section of Endocrinology, Department of Internal Medicine, Yale School of Medicine, New Haven, CT USA; 7 Section of Geriatrics, Department of Internal Medicine, Yale School of Medicine, New Haven, CT USA

**Keywords:** obesity, older adults, polypharmacy

## INTRODUCTION

Polypharmacy is highly prevalent among older adults and is associated with adverse drug events, treatment burden, and reduced quality of life.^1,2^ Obesity contributes to many of the chronic conditions that drive polypharmacy,^3,4^ but the extent of its contribution to polypharmacy has not been well quantified. In this study, we estimate the proportion of polypharmacy attributable to obesity in a nationally representative sample of U.S. older adults.

## METHODS

We performed a cross-sectional analysis using data from the 2021–2023 National Health and Nutrition Examination Survey (NHANES). We included adults aged ≥ 65 years with body mass index (BMI) ≥ 18.5 kg/m^2^ and excluded adults missing data on BMI or medication use. The independent variables were obesity, measured via BMI (≥ 30 kg/m^2^) or waist circumference (≥ 102 cm in men and ≥ 88 cm in women) and obesity class (class 1 = BMI 30 to < 35 kg/m^2^, class 2–4 = BMI ≥ 35 kg/m^2^). The dependent variable was polypharmacy, defined as self-reporting taking ≥ 5 prescription medications.

We compared those with and without obesity by age, sex, race, ethnicity, and clinical conditions. Categorical variables were compared using the χ^2^ test and continuous variables using the *t*-test. Using logistic regression, we calculated the adjusted relative risk of polypharmacy in participants with BMI-defined obesity compared to those without obesity, controlling for age, sex, education, smoking status (never, former, current), and alcohol use (not significant use, significant use, missing). We calculated the population attributable fraction (PAF) of polypharmacy due to BMI-defined obesity using this adjusted relative risk (aRR) and the prevalence of BMI-defined obesity among older adults with polypharmacy (P_1_) with the formula PAF = P_1_ * [(aRR – 1)/aRR] * 100.^5^ We additionally calculated the PAF of polypharmacy by obesity class and waist-circumference-defined obesity. Population-level estimates accounted for the complex survey design and were calculated using R version 4.2.3 (R Foundation).

Yale University institutional review board did not require review because this study did not involve human subjects. The study followed the Strengthening the Reporting of Observational Studies in Epidemiology (STROBE) reporting guideline.

## RESULTS

**Figure 1:**
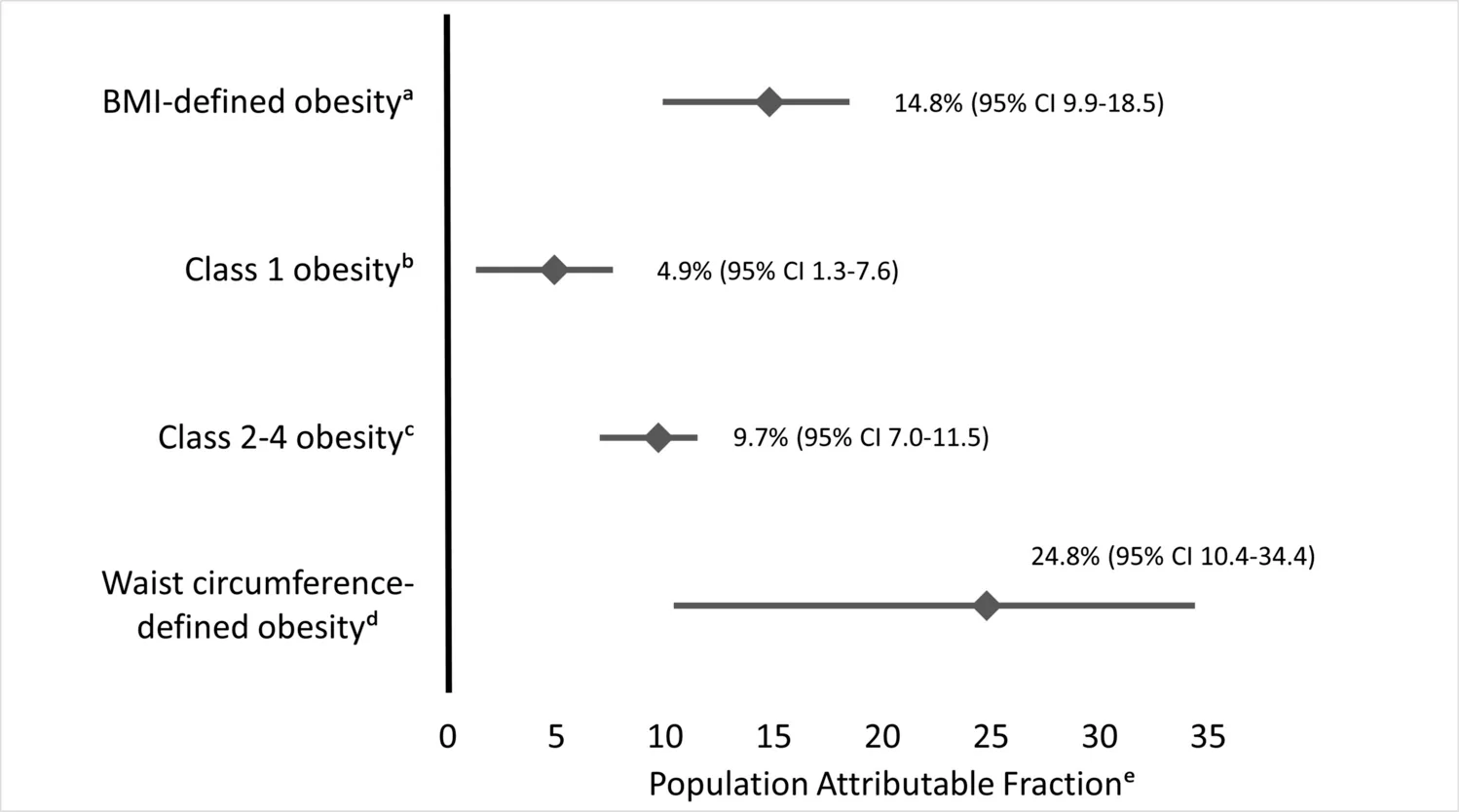
Population attributable fraction of polypharmacy attributable to BMI-defined obesity, class I obesity, class II obesity, and waist circumference-defined obesity. ^a^BMI-defined obesity is body mass index (BMI) ≥ 30 kg/m^2^. ^b^Class 1 obesity is BMI ≥ 30 kg/m^2^ and < 35 kg/m^2^. ^c^Class 2–4 obesity is BMI ≥ 35 kg/m^2^. ^d^Waist circumference-defined obesity is ≥ 1028 cm in men and ≥ 88 cm in women. ^e^The population attributable fraction (PAF) represents the proportion of polypharmacy cases (≥ 5 prescription medications) among older adults that is attributable to obesity, class 1 obesity, or class 2–4 obesity. A PAF with a 95% CI that does not cross zero is statistically significant. To calculate PAFs, logistic regression was used to obtain adjusted odds ratios, representing the odds of polypharmacy among the older adult population with obesity to the odds of polypharmacy among the older adult population without obesity, controlling for age, sex, education (high school or less, greater than high school), smoking status (never, former, current, missing), and significant alcohol intake (> 2 drinks per day on average over the past year for males or > 1 drink per day on average over the past year for females). The adjusted odds ratio was converted to an adjusted relative risk using the following formula: aRR = aOR / (1—P_0_ + (P_0_ * aOR)), where P_o_ is the proportion of older adults without obesity who had polypharmacy, aOR is the adjusted odds ratio, and aRR is the adjusted relative risk. The PAF was calculated using the following formula: PAF = proportion of older adults with polypharmacy who had obesity * [(aRR – 1)/aRR] * 100.^5^ The same method was used to calculate PAFs due to class 1 obesity and PAFs due to class 2 -4 obesity. All estimates are weighted to be representative of the US civilian, noninstitutionalized population.

The study sample included 1,944 individuals, representing 53.2 million older adults (weighted mean [SD] age, 72.7 [5.2] years; 55% female; 3.9% Asian, 8.7% Black, 8.2% Hispanic, 73.5% White, and 5.7% other or multiple races or ethnicities) (Table 1). Almost two-fifths (38.7%, 95% CI 35.9%-41.5%) had BMI-defined obesity (22.6% with class I obesity and 16.1% with classes 2–4 obesity), 70.5% (95% CI 67.7%-73.2%) had waist circumference-defined obesity. The prevalence of polypharmacy was 41.8% (95% CI 38.9%-44.7%), representing 22 million older adults. Polypharmacy was more prevalent among people with BMI-defined obesity compared to those without BMI-defined obesity (51.1% [95% CI 47.0%-55.1%] vs. 35.9% [95% CI 32.9%-39.0%]; p < 0.001). The PAF of polypharmacy due to BMI-defined obesity was 14.8% (9.9%-18.5%), corresponding to approximately 3.3 million older adults with polypharmacy attributable to BMI-defined obesity (Fig. 1). The PAF for polypharmacy due to class I obesity was 4.9% (95% CI 1.3%-7.6%), for class 2–4 obesity was 9.7% (95% CI 7.0%-11.5%), and for waist circumference-defined obesity was 24.8% (95% CI 10.4%-34.4%).

**Table 1:** Demographic and Clinical Characteristics of Older Adults by BMI-Defined Obesity, 2021-2023^a^

	**Total Population**	**Stratified by BMI-Defined Obesity**^**b**^		
		**No Obesity**	**Obesity**	***P*-value**
Unweighted No. of participants	1,944	1,179	765	NA
Weighted Population Size, millions (95% CI)	53.2 (46.0–60.4)	32.6 (27.4–37.9)	20.6 (18.1–23.1)	NA
Age mean, years (SD)	72.7 ± 5.2	73.0 ± 5.2	72.2 ± 5.0	0.030
Sex, % (95% CI)				
Female	55.0 (53.0–57.0)	54.6 (51.6–57.7)	55.5 (51.6–59.5)	0.760
Male	45.0 (43.0–47.0)	45.4 (42.3–48.4)	44.5 (40.5–48.4)	
Race and ethnicity^c^, % (95% CI)				
Hispanic	8.2 (4.2–12.1)	7.5 (3.9–11.2)	9.2 (4.3–14.1)	< 0.001
Non-Hispanic Asian	3.9 (2.4–5.5)	5.8 (3.4–8.3)	0.9 (0.2–1.7)	
Non-Hispanic Black	8.7 (5.4–12.0)	8.0 (5.1–10.9)	9.8 (5.6–14.0)	
Non-Hispanic White	73.5 (69.7–77.2)	72.9 (68.6–77.2)	74.4 (69.1–79.6)	
Other / Multiracial	5.7 (4.7–6.7)	5.7 (4.2–7.3)	5.7 (4.1–7.2)	
Clinical Conditions^d^, % (95% CI)				
Cardiovascular disease	24.9 (22.4–27.5)	22.2 (19.7–24.8)	29.2 (24.7–33.7)	0.004
Diabetes	25.2 (21.3–29.0)	19.1 (15.4–22.8)	34.8 (29.7–39.9)	< 0.001
Depression	8.3 (6.4–10.3)	6.7 (4.8–8.6)	10.8 (8.2–13.4)	< 0.001
Hypertension	64.3 (61.2–67.4)	58.7 (55.4–62.0)	73.2 (69.4–77.0)	< 0.001

Abbreviations: *NA*, not applicable

^a^All estimates are weighted to be representative of the US civilian, noninstitutionalized population, unless otherwise specified

^b^BMI-defined obesity is body mass index (BMI) ≥ 30 kg/m^2^

^c^Race and ethnicity were self-reported by NHANES participants. The other category includes other undefined non-Hispanic races, including non-Hispanic multiple race individuals

^d^Cardiovascular disease was defined as self-reported history of heart failure, stroke, myocardial infarction, angina, or coronary heart disease. Diabetes was defined as self-reported diagnosis of diabetes or HbA1c ≥ 6.5%. Depression was defined as a score of ≥ 10 on the Patient Health Questionnaire-9 (PHQ-9). Hypertension was defined as a systolic blood pressure ≥ 140, diastolic blood pressure ≥ 90, or self-reported of current blood pressure medication use

## CONCLUSIONS

In this cross-sectional study, we found that obesity accounted for between 1 in 7 (BMI-defined obesity) and 1 in 4 (waist circumference-defined obesity) cases of polypharmacy among older adults. Obesity medications which provide significant weight loss can improve many obesity-related complications (e.g., diabetes, sleep apnea)^6^ and may potentially reduce obesity-related polypharmacy. However, the potential benefit of reducing polypharmacy needs to be weighed against the immediate increase in medication burden and side effects. Future studies should examine the impact of medications for obesity on polypharmacy among older adults with obesity.

Limitations include reliance on self-reported number of medications taken. Older adults in long term care settings were not included in this study, limiting generalizability to that population. There may be unmeasured confounding, e.g., due to wealth or access to care, that impacts the association between obesity and polypharmacy. There may be important differences in PAF within subgroups, but the evaluation of these differences is beyond the scope of this brief report. Cross-sectional data limits our ability to determine a temporal relationship between obesity and polypharmacy, although longitudinal studies have demonstrated this relationship.^7^

These findings underscore obesity as a potentially modifiable contributor to polypharmacy in older adults and highlight the importance of considering medication burden when evaluating treatment.

## Data Availability

The datasets during and/or analyzed during the current study are available from CDC at https://wwwn.cdc.gov/nchs/nhanes/Default.aspx.
